# Clinical characteristics and risk factors for 90-day overall survival among 204 adult patients with secondary hemophagocytic lymphohistiocytosis: Experience from a single-center retrospective study

**DOI:** 10.3389/fmed.2022.774959

**Published:** 2022-10-10

**Authors:** Dongguang Wang, Xiang Tong, Sitong Liu, Wentao Zhang, Lian Wang, Shijie Zhang, Tianli Zhang, Qian Wang, Hong Fan

**Affiliations:** ^1^Department of Respiratory and Critical Care Medicine, West China Hospital/West China School of Medicine, Sichuan University, Chengdu, China; ^2^College of Computer Science, Sichuan University, Chengdu, China

**Keywords:** secondary hemophagocytic lymphohistiocytosis, adult, clinical characteristics, risk factors, prognostic model

## Abstract

**Objectives:**

To describe the clinical characteristics of secondary hemophagocytic lymphohistiocytosis (HLH) among adult patients, investigate its risk factors for 90-day overall survival (OS) from diagnosis, and establish a new prognostic model applicable to adult patients with secondary HLH.

**Methods:**

We conducted a retrospective cohort study of 204 adult patients with secondary HLH, between January 2010 and December 2020. All patients met at least five HLH-2004 criteria. Clinical features, laboratory results, treatments, and clinical outcomes of the patients were reviewed. Prognostic factors associated with 90-day overall survival from diagnosis were screened using Cox proportional hazard models.

**Results:**

The most common trigger was malignancy (61.3%). Multivariate analysis showed that age, coagulopathy, levels of hemoglobin, aspartate aminotransferase (AST), lactate dehydrogenase (LDH), creatinine, ferritin, and prothrombin time (PT) were independent prognostic factors for 90-day OS from the diagnosis of HLH. Based on the above risk factors, the patients were further divided into two groups: low-risk (≤4 risk factors) and high-risk (>4 risk factors), with overall 90-day survival rates of 82.7 and 28.1%, respectively (*P* < 0.001).

**Conclusion:**

Patients with older age, coagulopathy, lower hemoglobin, and AST levels, elevated LDH, creatinine and ferritin levels, and prolonged PT tended to have a worse prognosis. Moreover, our prognostic model provides the possibility of forecasting the clinical outcome of adult secondary HLH patients, although a larger sample, multicenter, randomized controlled clinical study is needed to verify the accuracy of the prognostic model.

## Introduction

Hemophagocytic lymphohistiocytosis (HLH), also known as hemophagocytic syndrome (HPS), is a rare, life-threatening, and systemic hyperinflammatory disorder that can rapidly deteriorate to disseminated intravascular coagulation (DIC), multiple organ failure, and death (1). Generally, the in-hospital mortality of HLH is reported to range from 20 to 75%, with an even greater risk of death in critically ill patients (2). It is clinically characterized by persistent high fever, cytopenia, hepatosplenomegaly, and activated macrophage infiltration into hematopoietic organs. According to the guidelines from the International Histiocyte Society, a diagnosis of HLH requires molecular evidence or the fulfillment of at least five out of eight HLH-2004 criteria (3): fever, splenomegaly, cytopenia, hypertriglyceridemia or hypofibrinogenemia, hyperferritinemia, elevated soluble interleukin-2 receptor (sIL-2R/sCD25), decreased or deficient natural killer cell activity, and hemophagocytosis in bone marrow, spleen, or lymph node biopsy. Traditionally, HLH is classified into primary (familial) and secondary (acquired) HLH, with secondary HLH accounting for ~90% of all affected patients (4). Compared to primary HLH which results from genetic defects, more underlying triggers have been identified in secondary HLH, including infections, malignancies, autoimmune diseases, acquired immunodeficiency, and drug therapies (1, 5). Recent studies suggest that malignancies and infections are the two main causes of secondary HLH, and epidemiological data from China show that ~60% of cases are caused by malignant neoplasms, especially hematological malignancies (6, 7).

HLH may occur at any age, although a nationwide survey of HLH in Japan indicated that over half of the patients were juveniles younger than 15 years of age (8). Over the past several decades, HLH has been well-described in pediatric patients; however, few scientific analyses of adult HLH have been performed, with most of them being case reports or series. Moreover, the existing literature on adult HLH predominantly focuses on diagnoses and clinical therapies, while only a few small studies provide prognostic data on adult HLH patients. Therefore, we performed a retrospective cohort study on 204 adult patients diagnosed with secondary HLH, to summarize their clinical characteristics, identify the risk factors associated with 90-day overall survival (OS) from diagnosis, stratify patients with all independent risk factors, and preliminarily establish a prognostic model to improve the accuracy of prognosis prediction in adult patients with secondary HLH.

## Materials and methods

### Study population

A retrospective cohort study involving 204 consecutively diagnosed adult patients with HLH (114 male and 90 female) was conducted at the West China Hospital of Sichuan University in Chengdu, China, from January 2010 to December 2020. The eligibility criteria were as follows: (1) patients aged > 18 years and (2) fulfillment of five or more of the eight HLH-2004 diagnostic criteria. Patients who met any of the following criteria were excluded: (1) genetic abnormalities suspected to be primary HLH; (2) the same patient readmitted for recurrent HLH; (3) patients with primary or acquired immunodeficiency disorders; and (4) patients lost to follow-up within 90 days of HLH diagnosis.

An initial diagnosis of secondary HLH was established based on previously proposed clinical guidelines (HLH-2004) (3): (1) fever; (2) splenomegaly; (3) two or more cell lineages of cytopenia (hemoglobin < 90 g/L, platelets < 100 × 10^9^/L, neutrophils < 1.0 × 10^9^/L); (4) hypertriglyceridemia (triglyceride > 3.0 mmol/L) or hypofibrinogenia (fibrinogen < 1.5 g/L); (5) hemophagocytosis found in the bone marrow, spleen, or lymph nodes; (6) hyperferritinemia (ferritin ≥ 500 μg/L); (7) elevated concentration of soluble interleukin 2 receptor (sIL-2R/sCD25 ≥ 2,400 u/mL); and (8) low or absent activity of natural killer (NK) cells. Because NK cell activity detection was not available in our hospital, the diagnosis was based on the other seven diagnostic criteria.

### Data collection and processing

The medical records of each patient were reviewed and demographic and clinical data were extracted and addressed using a predesigned template. Generally, the medical information collected for this study included demographic data (sex and age), clinical characteristics (highest recorded body temperature, clinical manifestations, presence of organ enlargement confirmed by sonographic or tomographic assessment, and underlying triggering factors), laboratory parameters [Epstein-Barr virus loads, hemoglobin levels, platelet counts, leukocyte counts, serum bilirubin levels, hepatic enzyme levels, lactate dehydrogenase (LDH) levels, albumin levels, blood urea nitrogen (BUN) levels, creatinine levels, triglyceride (TG) levels, sodium and calcium levels, prothrombin time (PT), fibrinogen levels, ferritin concentrations, and sIL-2R/sCD25 concentrations], hemophagocytosis confirmed by bone marrow biopsies, and clinical outcome within 90 days after diagnosis of secondary HLH. The laboratory results extracted from the medical records were those performed on the day of diagnosis with HLH or up to 3 days before or after in the absence of data from the same day.

The clinical outcomes of patients with secondary HLH within 90 days of diagnosis were confirmed by their hospital clinical records or by telephone calls to the patients or their family members.

### Statistical analysis

Quantitative data in accordance with normal distribution were presented as mean ± standard deviation (SD), and differences between groups were compared using Student's *t*-test; a non-parametric Mann–Whitney *U*-test was used for skewness distribution data, indicated as medians [interquartile range (IQR)]. Categorical variables were presented as numbers and proportions, and chi-square or Fisher's exact tests were used to compare intergroup differences. The optimal cutoff value of continuous variables and their sensitivity and specificity were determined using receiver operating characteristic (ROC) curves and maximum Youden's index. OS was defined as the time from the diagnosis of secondary HLH to death from any cause. The Kaplan-Meier method with a log-rank test was used for OS assessment. Univariate and multivariate analyses were performed using a Cox proportional hazards model. Statistical significance was defined as a two-tailed *P*-value < 0.05. All analyses were performed using the SPSS software (version 22.0; IBM Corp., Armonk, NY, USA) or the STATA software (version 12.0; Stata Corp., College Station, TX, USA).

## Results

### General characteristics of 204 adult patients diagnosed with secondary HLH

Data of 448 patients diagnosed with HLH and discharged between January 2010 and December 2020 were retrieved from the database of the West China Hospital of Sichuan University. As shown in Supplementary Figure 1, 204 adult patients with secondary HLH were included in the study. Of these, 114 (55.9%) were male and 90 (44.1%) were female, with an overall median age of 44.00 (29.00, 56.00) years (Table 1). According to the HLH-2004 diagnostic criteria, 191 (93.6%) patients had a prolonged fever of ≥38.5°C, and splenomegaly was found in 173 (84.8%) patients. Two or more lines of cytopenia occurred in 174 (85.3%) patients, while 164 had elevated triglyceride or decreased fibrinogen levels. All 204 patients underwent bone marrow biopsy; among them, only 87 (42.6%) were histopathologically found to have hemophagocytosis. Elevated serum levels of ferritin and sIL-2R/sCD25 were observed in most patients, with 194 of 196 (99.0%) for ferritin and 128 of 137 (93.4%) for sIL-2R/sCD25 (Supplementary materials).

**Table 1:** Demographic and clinical characteristics among patients with secondary HLH.

	**All (*n* = 204)**	**Survivors (*n* = 134)**	**Non-survivors (*n* = 70)**	**P value**
**Demographical characteristics**				
Gender (male/female)	114 (55.9)/90 (44.1)	76 (56.7)/58 (43.3)	38 (54.3)/32 (45.7)	0.740
Age (years)	44.00 (29.00, 56.00)	45.50 (27.75, 56.00)	40.50 (29.00, 55.00)	0.934
**Underlying triggering factor**				
Infections	65 (31.9)	44 (32.8)	21 (30.0)	0.660
Malignancies	125 (61.3)	79 (59.0)	46 (65.7)	
Autoimmune disorder	8 (3.9)	6 (4.5)	2 (2.9)	
Unknown	6 (2.9)	5 (3.7)	1 (1.4)	
**Clinical manifestations**				
Respiratory system	110 (53.9)	70 (52.2)	40 (57.1)	0.505
Digestive system	73 (35.8)	48 (35.8)	25 (35.7)	0.988
Neural system	80 (39.2)	51 (38.1)	29 (41.4)	0.640
**Organ enlargement**				
Liver	74 (36.3)	53 (39.6)	21 (30.0)	0.178
Spleen	173 (84.8)	120 (89.6)	53 (75.7)	0.009^*^
Lymph nodes	124 (60.8)	81 (60.4)	43 (61.4)	0.892
Skin rashes	27 (13.2)	17 (12.7)	10 (14.3)	0.749
Jaundice	24 (11.8)	14 (10.4)	10 (14.3)	0.419
Coagulopathy	53 (26.0)	26 (19.4)	27 (38.6)	0.003^*^
**Laboratory examinations**				
EBV-DNA ≥10^2^ copies/mL	128 (62.7)	84 (62.7)	44 (62.9)	1.000
Hemoglobin (g/L)	78.00 (67.00, 89.75)	82.00 (70.75, 93.25)	72.00 (61.00, 81.25)	<0.001^*^
Platelet ( × 10^9^/L)	39.00 (18.00, 65.00)	45.00 (25.00, 77.50)	29.00 (14.00, 47.25)	<0.001^*^
Leukocyte ( × 10^9^/L)	1.87 (1.07, 4.01)	2.03 (1.23, 4.60)	1.41 (0.88, 3.09)	0.461
Neutrophil ( × 10^9^/L)	1.25 (0.61, 2.82)	1.36 (0.68, 3.31)	0.98 (0.40, 2.06)	0.213
Lymphocyte ( × 10^9^/L)	0.42 (0.22, 0.76)	0.47 (0.25, 0.76)	0.31 (0.20, 0.75)	0.631
Monocyte ( × 10^9^/L)	0.10 (0.05, 0.23)	0.11 (0.05, 0.28)	0.08 (0.03, 0.20)	0.976
TB (μmol/L)	18.75 (11.73, 44.10)	16.45 (10.45, 34.70)	27.25 (15.90, 82.08)	0.007^*^
ALB (g/L)	27.55 (24.33, 31.00)	28.25 (24.80, 31.80)	26.30 (22.88, 30.15)	0.007^*^
ALT (IU/L)	64.50 (28.50, 153.50)	54.00 (27.75, 141.00)	75.50 (32.50, 169.00)	0.092
AST (IU/L)	91.00 (44.25, 230.75)	80.50 (38.00, 197.00)	133.50 (54.00, 373.75)	0.089
LDH (IU/L)	742.00 (480.50, 1263.25)	617.50 (390.75, 1099.00)	905.00 (639.75, 1607.50)	0.033^*^
GGT (IU/L)	139.00 (55.00, 262.50)	101.00 (44.75, 232.00)	160.00 (77.50, 302.00)	0.196
BUN (mmol/L)	5.58 (3.97, 7.90)	4.83 (3.78, 7.43)	6.28 (4.68, 8.58)	0.006^*^
Creatinine (μmol/L)	57.55 (46.25, 74.00)	57.05 (44.75, 72.00)	60.50 (47.00, 81.25)	0.066
TG (mmol/L)	2.72 (1.76, 3.88)	2.34 (1.65, 3.71)	3.09 (2.16, 4.41)	0.015^*^
Na^+^ (mmol/L)	134.35 (131.63, 137.85)	134.35 (131.88, 137.28)	134.20 (130.53, 138.45)	0.823
Ca^2+^ (mmol/L)	1.92 (1.78, 2.03)	1.94 (1.81, 2.03)	1.88 (1.74, 2.03)	0.089
PT (s)	13.85 (12.20, 16.65)	13.40 (11.98, 15.23)	15.15 (12.70, 18.83)	0.075
FIB (g/L)	1.23 (0.80, 1.91)	1.39 (0.90, 2.36)	0.95 (0.64, 1.29)	0.002^*^
CRP (mg/L)	35.30 (17.05, 79.08)	30.80 (16.15, 77.15)	41.60 (20.10, 88.80)	0.372
Ferritin (≥500 μg/L)	194/196 (99.0)	129/131 (98.5)	65/65 (100.0)	1.000
sIL-2R/sCD25 (≥2,400 U/mL)	128/137 (93.4)	89/95 (93.7)	39/42 (92.9)	1.000
Hemophagocytosis	87 (42.6)	59 (44.0)	28 (40.0)	0.655
Therapeutic regimen (based on dexamethasone and etoposide)	73 (35.8)	51 (38.1)	22 (31.4)	0.348

* P < 0.05.

EBV-DNA, Epstein-Barr virus deoxyribonucleic acid; TB, total bilirubin; ALB, albumin; ALT, alanine aminotransferase; AST, aspartate aminotransferase; LDH, lactate dehydrogenase; GGT, γ-glutamyl transpeptidase; BUN, blood urea nitrogen; TG, triglyceride; PT, prothrombin time; FIB, fibrinogen; CRP, C-reactive protein; sIL-2R/sCD25, soluble interleukin-2 receptor.

The laboratory results were those obtained on the day of diagnosis of hemophagocytic lymphohistiocytosis, or those obtained up to 3 days before or after in the absence of data from the same day. The bold values in tables mean statistically significant *P* values.

Malignancies were the most common underlying triggering factor, accounting for 61.3% of all cases, followed by infections (31.9%), and autoimmune disorders (3.9%). However, the triggers of HLH in six (2.9%) patients remained unclear until their discharge from the hospital. The clinical symptoms of adult HLH patients were also collected in this study, of which 110 (53.9%) patients had symptoms in the respiratory system, 73 (35.8%) in the digestive system, and 80 (39.2%) in the neural system. Organ enlargement might be a helpful clue for hemophagocytic lymphohistiocytosis, with 84.8% of patients having splenomegaly, 60.8% having enlarged lymph nodes, and 36.3% having hepatomegaly on ultrasonic or radiological examinations. In addition, a small number of patients in our study had skin rashes (13.2%), jaundice (11.8%), or coagulopathy (26.0%).

In terms of laboratory results, high loads of EBV-DNA (≥10^2^ copies/mL) in the peripheral blood were found in 128 (62.7%) patients. Additionally, abnormal hematological results of blood cell counts and coagulation function tests in adult secondary HLH patients were found, with a median hemoglobin level of 78.00 (67.00, 89.75) g/L, median leukocyte count of 1.87 (1.07, 4.01) × 10^9^ /L, median platelet count of 39 (18, 65) × 10^9^ /L, median prothrombin time of 13.85 (12.20, 16.65) s, and median fibrinogen level of 1.23 (0.80, 1.91) g/L. Interestingly, we also found decreased serum albumin levels among patients with HLH in the present study, with a median albumin level of 27.55 (24.33, 31.00) g/L.

Seventy-three (35.8%) patients received a therapeutic regimen consisting of at least dexamethasone and etoposide, and 60 (29.4%) received an etiological treatment only. The detailed demographic and clinical data are presented in Table 1.

### Comparison of clinical and laboratory findings between survivors and non-survivors

In this study, 134 (65.7%) patients survived at the time of follow-up and 70 (34.3%) died. Interestingly, survivors seemed more likely to have splenomegaly (*P* = 0.009), while more cases in the non-survivor group had coagulopathy, such as epistaxis, subcutaneous ecchymosis, and gastrointestinal hemorrhage (*P* = 0.003). In contrast to the survivors, non-survivors also showed lower hemoglobin (*P* < 0.001), platelet (*P* < 0.001), albumin (*P* = 0.007), and fibrinogen (*P* = 0.002) levels, and higher levels of total bilirubin (*P* = 0.007), LDH (*P* = 0.033), BUN (*P* = 0.006), and TG (*P* = 0.015) (Table 1).

### Determination of optimal cut-off values of continuous clinical and laboratory variables

ROC analyses and the maximum Youden's index were used to determine the most appropriate cutoff value of continuous variables for the 90-day OS in adult patients with secondary HLH. Table 2 displays the results of the cutoff values of continuous clinical and laboratory variables.

**Table 2:** The optimal cutoff value of continuous clinical and laboratory variables based on ROC analyses and maximum Youden's index.

**Variables**	**Cut-off value**	**AUC**	**95%CI**	**Sensitivity**	**Specificity**
Age (years)	25	0.501	0.419–0.583	0.900	0.209
Hemoglobin (g/L)	82	0.674	0.597–0.751	0.530	0.757
Platelet (× 10^9^/L)	33	0.650	0.571–0.728	0.664	0.586
Leukocyte (× 10^9^/L)	1.37	0.601	0.517–0.685	0.724	0.500
Neutrophil (× 10^9^/L)	0.61	0.594	0.511–0.678	0.806	0.371
Lymphocyte (× 10^9^/L)	0.30	0.555	0.470–0.640	0.687	0.486
Monocyte (× 10^9^/L)	0.06	0.568	0.483–0.654	0.739	0.414
TB (μmol/L)	17.5	0.658	0.581–0.735	0.743	0.552
ALB (g/L)	23.3	0.601	0.518–0.683	0.873	0.314
ALT (IU/L)	71	0.555	0.470–0.639	0.557	0.582
AST (IU/L)	119	0.608	0.526–0.690	0.557	0.634
LDH (IU/L)	687	0.661	0.584–0.739	0.743	0.575
GGT (IU/L)	114	0.600	0.521–0.680	0.714	0.530
BUN (mmol/L)	4.8	0.641	0.563–0.718	0.743	0.493
Creatinine (μmol/L)	118	0.542	0.456–0.628	0.143	0.978
TG (mmol/L)	2.17	0.626	0.547–0.704	0.757	0.478
Na^+^ (mmol/L)	130.1	0.510	0.422–0.598	0.873	0.243
Ca^2+^ (mmol/L)	1.86	0.577	0.490–0.663	0.716	0.471
PT (s)	17.1	0.653	0.572–0.735	0.429	0.888
FIB (g/L)	1.10	0.697	0.621–0.773	0.679	0.657

AUC, area under the curve; CI, confidence interval; TB, total bilirubin; ALB, albumin; ALT, alanine aminotransferase; AST, aspartate aminotransferase; LDH, lactate dehydrogenase; GGT, γ-glutamyl transpeptidase; BUN, blood urea nitrogen; TG, triglyceride; PT, prothrombin time; FIB, fibrinogen.

The laboratory results were those obtained on the day of diagnosis of hemophagocytic lymphohistiocytosis, or those obtained up to 3 days before or after in the absence of data from the same day.

### Risk factors associated with 90-day OS for adult secondary HLH

**Figure 1:**
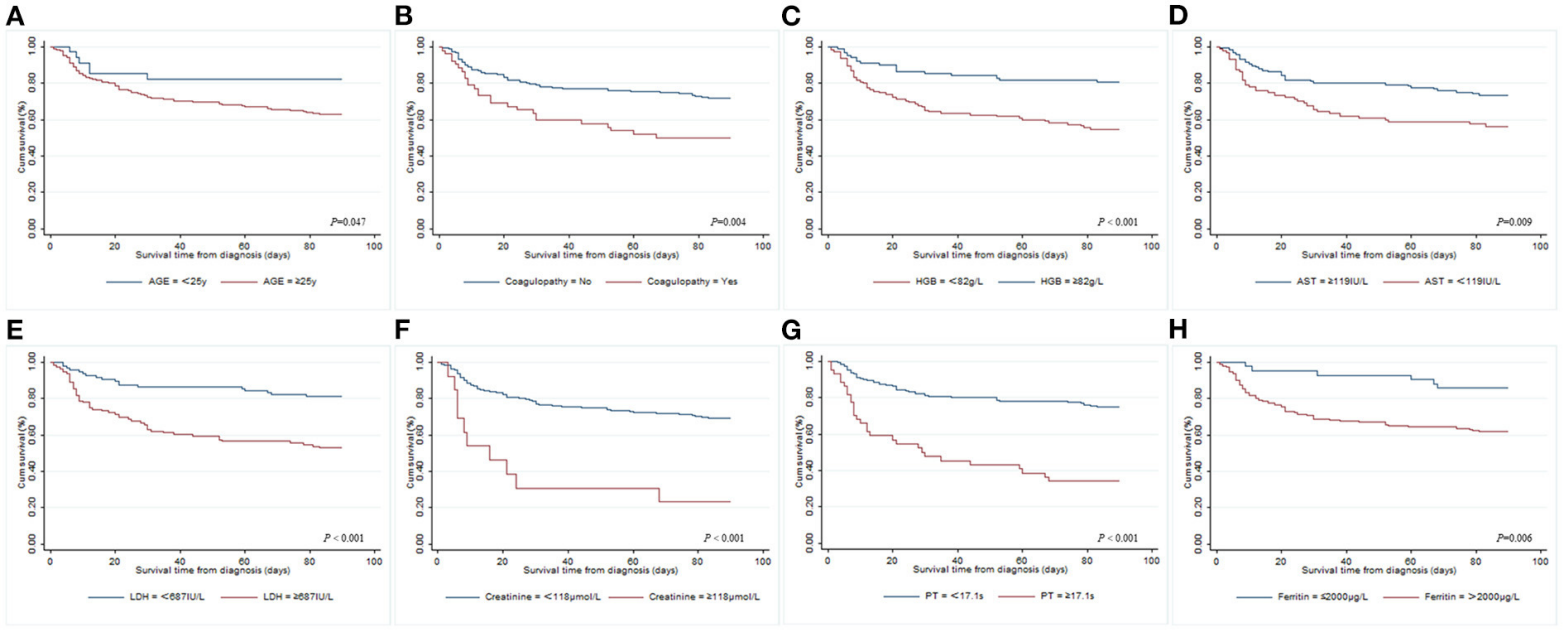
Kaplan–Meier survival curves of adult patients with different risk factors for 90-day overall survival from diagnosis of secondary HLH. **(A)** Age; **(B)** coagulopathy; **(C)** hemoglobin; **(D)** AST; **(E)** LDH; **(F)** creatinine; **(G)** PT; **(H)** ferritin.

Univariate and multivariate Cox regression models were used to determine independent risk factors associated with 90-day OS among adult patients with secondary HLH. A total of 196 cases were included after excluding missing values. As shown in Table 3, 23 significant variables were identified by univariate Cox regression analysis, while only age ≥25 years old (HR 2.435, 95% CI 1.004–5.908, *P* = 0.049), coagulopathy (HR 2.517, 95% CI 1.406–4.509, *P* = 0.002), hemoglobin ≥ 82.0 g/L (HR 0.321, 95% CI 0.162–0.634, *P* = 0.001), aspartate aminotransferase (AST) ≥ 119 IU/L (HR 0.405, 95% CI 0.169–0.967, *P* = 0.042), LDH ≥ 687 IU/L (HR 2.604, 95% CI 1.173–5.784, *P* = 0.019), creatinine ≥ 118 μmol/L (HR 4.127, 95% CI 1.708–9.972, *P* = 0.002), PT ≥ 17.1 s (HR 2.346, 95% CI 1.212–4.542, *P* = 0.011), and ferritin > 2,000 μg/L (HR 3.346, 95% CI 1.103–10.145, *P* = 0.033) were found to be statistically significant by multivariate Cox analysis. The survival curves of adult patients with secondary HLH based on significant risk factors for 90-day OS are shown in Figure 1.

**Table 3:** Risk factors for 90-day overall survival by univariate and multivariate Cox regression.

**Variables**	**Univariate**			**Multivariate**		
	**Hazard ratio**	**95% CI**	***P*** **-value**	**Hazard ratio**	**95% CI**	**P value**
Age (years)	2.338	1.012–5.402	0.047^*^	2.435	1.004–5.908	0.049^*^
Splenomegaly	0.427	0.247–0.739	0.002^*^	0.559	0.276–1.130	0.105
Coagulopathy	2.062	1.266–3.358	0.004^*^	2.517	1.406–4.509	0.002^*^
Hemoglobin (g/L)	0.362	0.209–0.626	<0.001^*^	0.321	0.162–0.634	0.001^*^
Platelet (× 10^9^/L)	0.427	0.265–0.690	0.001^*^	1.498	0.780–2.876	0.225
Leukocyte (× 10^9^/L)	0.465	0.290–0.746	0.001^*^	0.592	0.278–1.263	0.175
Neutrophil (× 10^9^/L)	0.529	0.325–0.862	0.011^*^	0.959	0.441–2.081	0.915
Lymphocyte (× 10^9^/L)	0.530	0.331–0.850	0.008^*^	0.544	0.292–1.016	0.056
Monocyte (× 10^9^/L)	0.557	0.345–0.899	0.017^*^	1.215	0.632–2.335	0.559
TB (μmol/L)	3.044	1.759–5.268	<0.001^*^	2.117	0.983–4.557	0.055
ALB (g/L)	0.409	0.246–0.679	0.001^*^	0.851	0.376–1.926	0.699
ALT (IU/L)	1.660	1.031–2.673	0.037^*^	1.040	0.472–2.292	0.922
AST (IU/L)	1.878	1.168–3.019	0.009^*^	0.405	0.169–0.967	0.042^*^
LDH (IU/L)	3.046	1.778–5.217	<0.001^*^	2.604	1.173–5.784	0.019^*^
GGT (IU/L)	2.395	1.423–4.031	0.001^*^	1.627	0.834–3.175	0.154
BUN (mmol/L)	2.295	1.340–3.930	0.002^*^	1.077	0.530–2.187	0.838
Creatinine (μmol/L)	4.018	2.046–7.893	<0.001^*^	4.127	1.708–9.972	0.002^*^
TG (mmol/L)	2.518	1.439–4.406	0.001^*^	1.129	0.587–2.173	0.717
Na^+^ (mmol/L)	0.519	0.300–0.897	0.019^*^	0.499	0.232–1.074	0.075
Ca^2+^ (mmol/L)	0.535	0.333–0.858	0.010^*^	1.797	0.893–3.618	0.101
PT (s)	3.719	2.299–6.015	<0.001^*^	2.346	1.212–4.542	0.011^*^
FIB (g/L)	0.327	0.199–0.537	<0.001^*^	0.673	0.341–1.327	0.253
Ferritin (>2,000 μg/L)	3.242	1.399–7.511	0.006^*^	3.346	1.103–10.145	0.033^*^

* P < 0.05.

CI, confidence interval; EBV-DNA, Epstein-Barr virus deoxyribonucleic acid; TB, total bilirubin; ALB, albumin; ALT, alanine aminotransferase; AST, aspartate aminotransferase; LDH, lactate dehydrogenase; GGT, γ-glutamyl transpeptidase; BUN, blood urea nitrogen; TG, triglyceride; PT, prothrombin time; FIB, fibrinogen.

The laboratory results were those obtained on the day of diagnosis of hemophagocytic lymphohistiocytosis, or those obtained up to 3 days before or after in the absence of data from the same day. The bold values in tables mean statistically significant *P* values.

### Establishment of a risk model for 90-day overall survival among adult secondary HLH patients

**Figure 2:**
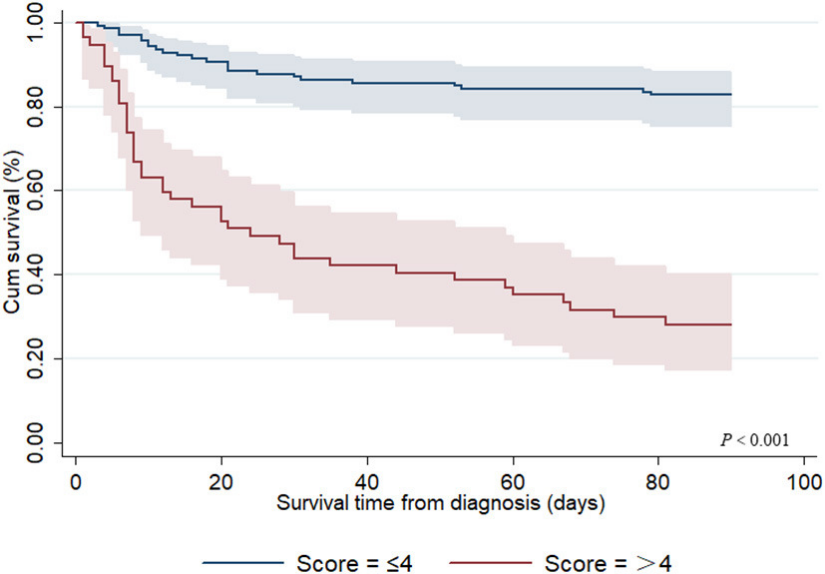
Kaplan–Meier survival curve for 90-day overall survival from diagnosis among adult secondary HLH patients with different risk scores.

A risk model for 90-day OS among adult patients with HLH was established based on the risk factors identified by Cox regression analyses, with one point given if a patient had any of the following risk factors: age ≥ 25 years old, coagulopathy, hemoglobin < 82.0 g/L, AST < 119 IU/L, LDH ≥ 687 IU/L, creatinine ≥ 118 μmol/L, PT ≥ 17.1 s, and ferritin > 2,000 μg/L. The total score was calculated using the number of risk factors, with a range of 0–8. Thus, in our study, three (1.5%) patients scored 1, 23 (11.7%) scored 2, 52 (26.5%) scored 3, 61 (31.1%) scored 4, 40 (20.4%) scored 5, 16 (8.2%) scored 6, and 1 (0.5%) scored 7, and none of the patients scored 0 or 8. Subsequently, statistical differences among the groups with different scores were tested, and the results showed that there were no differences between the groups with scores 1 and 2, 2 and 3, 3 and 4, 5 and 6, and 6 and 7 (*P* = 1.000, *P* = 0.082*, P* = 0.345, *P* = 0.175, and *P* = 1.000, respectively); therefore, the patients could be further divided into two groups: low-risk (score ≤ 4) and high-risk (score > 4). As shown in Figure 2, the 90-day OS rate in the low-risk group was 82.7%, and 28.1% in the high-risk group (*P* < 0.001).

## Discussion

In this pilot study, we described the clinical characteristics of secondary HLH among adult patients and explored the risk factors associated with 90-day OS from diagnosis. HLH is a rapidly progressive, life-threatening systemic inflammatory syndrome characterized by excessive immune activation and an uncontrolled cytokine storm that results in myelosuppression and vascular endothelial injury (9). In total, the crude mortality rate in our study was 34.3%, with a median survival of 12.5 days among dead patients. A previous study conducted in 21 countries showed that >10% of pediatric patients died within 2 months of diagnosis due to coagulopathy in the visceral organs, opportunistic infections, or multiple organ failure (10). In contrast to adolescents, the prognosis is worse in adult patients. In their study, Otrock et al. reported that the 30-day mortality of adult HLH from admission was 30%, with the duration of hospital stays ranging from 1 to 89 days (11). In another systematic review of critically ill adult patients with HLH, the overall mortality was 57.8%, and the median time until death in the subgroup analysis was 26 days (IQR 10–73 days) (12). Although there has been a greater understanding of the pathogenesis of HLH over the past few decades, it is difficult to diagnose, and the treatment is often delayed due to the rarity, diversity, and complexity of the disease, resulting in the natural history of HLH being almost invariably fatal.

The reported predominant cause of HLH differs in countries, with infections being the main trigger of adult HLH in the USA, France, Spain, and South Korea, with malignancies being the most common in Italy, China, and Japan, suggesting a specific geographical distribution of triggering factors (13). Consistent with previous studies (14–16), we found that malignancies, especially lymphoma and leukemia, were the most common triggers of adult secondary HLH. In another study, infections were reported to be the most common cause of HLH (17). A multicenter retrospective etiological analysis of 601 HLH patients in China found that the most common causes of HLH were infections in children, infections and malignancies in youths, and malignancies in middle-aged and elderly groups (18). In summary, the diversity of age and geographical distribution might partially explain the differences in triggering factors among studies.

The clinical and laboratory characteristics of secondary HLH reflect tissue infiltration of activated immune cells, especially CD8^+^ T lymphocytes and macrophages, as well as local and systematic effects of various inflammatory cytokines, such as interferon-γ, tumor necrosis factor-α, interleukin-1β, and interleukin-6 (19). Several studies have reported multiple risk factors that correlate with the prognosis of secondary HLH in adult patients. In the present study, we found that age ≥25 years old, coagulopathy, lower levels of hemoglobin (< 82.0 g/L) and aspartate aminotransferase (< 119 IU/L), higher levels of lactate dehydrogenase (≥687 IU/L), creatinine (≥118 μmol/L), and ferritin (>2,000 μg/L), and prolonged prothrombin time (≥17.1 s) might be independent risk factors for 90-day OS from diagnosis. Additionally, in another study, blood-based inflammatory markers, such as lymphocyte-to-monocyte ratio (LMR) and red blood cell distribution width-to-platelet ratio (RPR) were considered independent factors for predicting the OS of patients with HLH (15). A prospective study of 20 lymphoma-associated patients with HLH revealed that the interferon-γ secretion capacity of lymphocytes was significantly decreased and might be used as a predictor of prognosis (20). From an etiopathically driven analysis of adult HLH, researchers found that infection with more than one microbiological agent was the only independent variable associated with mortality (21). In addition to these traditional predictors, novel biomarkers, such as soluble CD163, plasma pentraxin 3 (PTX3), and presepsin are critical for the diagnosis and prognosis of patients with secondary HLH (22–24). Although several laboratory parameters correlate with poor clinical outcomes of HLH, none of them have been widely used in clinical practice so far, which reflects the complicated nature of the disease to some extent. As a result, dynamic monitoring of clinical indicators, other than finding new predictive markers, appears to be more important.

Predictive models of death can dynamically evaluate the clinical conditions of critically ill patients and might be more in line with the needs of clinicians. However, few studies have established prognostic models for HLH. Zhou et al. developed a prognostic model for adult HLH based on a combination of three laboratory markers (ferritin, platelets, and alanine aminotransferase), which showed an obvious improvement in discriminating the risk of death in contrast with any of these markers alone (17). Meanwhile, in their study, a 0.412 cutoff reached a sensitivity of 76.9% and specificity of 78.9%. In another retrospective study from Korea, researchers established a prognostic model based on four parameters: age (2 points), EBV association (1 point), platelet count (1 point), and hyperferritinemia (1 point). The experimental results showed that the prognostic model had high discriminability for risk of death, with the 5-year OS rates in low- (score 0–1), intermediate- (score 2), and poor-risk (score ≥ 3) patients being 92.1, 36.8, and 18.0%, respectively (*P* < 0.001) (25). Considering the rapid progress of HLH and short median survival days, we developed a novel prognostic model for 90-day OS from the diagnosis of HLH. Based on our scoring system, the 90-day OS in the low-risk groups (score ≤ 4) was 82.7% and 28.1% in the high-risk group (score > 4) (*P* < 0.001). However, this study failed to verify the accuracy of the model in new groups. External validation is the optimal strategy for controlling the reliability of the predictive models. Unfortunately, this validation strategy is practically impossible to apply because HLH is a rare disease.

Our study had some limitations. First, due to the intrinsic defectiveness of retrospective studies, we had to accept some missing clinical and follow-up data. To avoid this, we did not use variables with missing values over 30% when building regression models, and patients without follow-up data were excluded at the beginning of the research. In addition, treatment and several important inflammatory biomarkers, such as sIL-2R/sCD25 and CRP, were not included in the analyses, which might have affected the precision of the model. Second, the maximum Youden's index was used to determine the optimal cutoff of the predictive model in this study, assigning equal weights to sensitivity and specificity; however, it was reported that weighted methods might achieve more outstanding discrimination abilities (26). Third, over 90% of patients in the present study had a trigger of malignancies or infections, indicating that caution is needed when applying this predictive model. Finally, to better clarify the terminology regarding HLH, some scholars proposed the concept of HLH disease mimics a few years ago, which referred to disorders leading to the HLH pattern, but not likely to benefit from immunosuppressive therapies (27). To a certain extent, this is conducive to the timely and targeted treatment of HLH. Unfortunately, we could not further distinguish HLH mimics from HLH syndrome in the current retrospective study, and thus, the concept of HLH mentioned in this paper is a broader syndrome of HLH. Considering the small number of studies on forecasting models, this study is still of great value in proposing a novel prognostic model for adult patients with secondary HLH. Multicenter prospective randomized controlled trials with larger sample sizes are needed to verify the precision of our predictive model.

## Conclusion

The current study described the clinical characteristics of adult patients with secondary HLH, explored the risk factors associated with 90-day OS, and established a risk model for the clinical outcomes of adult patients within 90 days of diagnosis. These results may help clinicians evaluate the prognosis of adult patients with secondary HLH, although a multicenter, randomized controlled clinical study with a larger sample size is needed to confirm this.

## Data availability statement

The original contributions presented in the study are included in the article/Supplementary material, further inquiries can be directed to the corresponding author/s.

## Ethics statement

This study was conducted in accordance with the Declaration of Helsinki and authorized by the institutional ethics board. In addition, the observational design of the study did not impose the need to obtain informed consent from individual patients.

## Author contributions

Study design and manuscript drafting: DGW, STL, and XT. Clinical data collection: DGW, LW, SZ, TZ, and QW. Data analyses: DGW and WZ. Manuscript revision: XT and HF. All authors have read the manuscript and approved the final manuscript.

## Funding

This work was supported by the National Key R&D Program of China (2017YFC1309703) and the 1.3.5 project for disciplines of excellence-Clinical Research Incubation Project, West China Hospital, Sichuan University (2019HXFH008).

## Conflict of interest

The authors declare that the research was conducted in the absence of any commercial or financial relationships that could be construed as a potential conflict of interest.

## Publisher's note

All claims expressed in this article are solely those of the authors and do not necessarily represent those of their affiliated organizations, or those of the publisher, the editors and the reviewers. Any product that may be evaluated in this article, or claim that may be made by its manufacturer, is not guaranteed or endorsed by the publisher.

## Supplementary material

Supplementary Figure 1 Flow chart of included patients and the simplified model.

Supplementary Table 1 Comparison of clinical characteristics between survivors and non-survivors of secondary HLH with different causes.

Supplementary Table 2 Clinical and laboratory findings of included secondary HLH patients according to the HLH-2004 criteria.

## Abbreviations

HLH: hemophagocytic lymphohistiocytosis
HPS: hemophagocytic syndrome
DIC: disseminated intravascular coagulation
CRP: C-reactive protein
sIL-2R/sCD25: soluble interleukin-2 receptor
NK cell: natural killer cell
AST: aspartate aminotransferase
LDH: lactate dehydrogenase
BUN: blood urea nitrogen
TG: triglyceride
PT: prothrombin time
SD: standard deviation
IQR: interquartile range
ROC: receiver operating characteristic
OS: overall survival
HR: hazard ratio
CI: confidence interval
EBV-DNA: Epstein-Barr virus deoxyribonucleic acid
LMR: lymphocyte-to-monocyte ratio
RPR: red blood cell distribution width-to-platelet ratio
PTX3: plasma pentraxin 3.

## References

[1] Morimoto A Nakazawa Y Ishii E. Hemophagocytic lymphohistiocytosis: pathogenesis, diagnosis, and management. Pediatr Int. (2016) 58:817–25. 10.1111/ped.13064 27289085

[2] Kapoor S Morgan CK Siddique MA Guntupalli KK. Intensive care unit complications and outcomes of adult patients with hemophagocytic lymphohistiocytosis: a retrospective study of 16 cases. World J Crit Care Med. (2018) 7:73–83. 10.5492/wjccm.v7.i6.73 30596029 PMC6305525

[3] Henter JI Horne A Arico M Egeler RM Filipovich AH Imashuku S. HLH-2004: Diagnostic and therapeutic guidelines for hemophagocytic lymphohistiocytosis. Pediatr Blood Cancer. (2007) 48:124–31. 10.1002/pbc.21039 16937360

[4] Lai W Wang Y Wang J Wu L Jin Z Wang Z. Epstein-Barr virus-associated hemophagocytic lymphohistiocytosis in adults and adolescents-a life-threatening disease: analysis of 133 cases from a single center. Hematology. (2018) 23:810–6. 10.1080/10245332.2018.1491093 29957156

[5] Hayden A Park S Giustini D Lee AY Chen LY. Hemophagocytic syndromes (HPSs) including hemophagocytic lymphohistiocytosis (HLH) in adults: a systematic scoping review. Blood Rev. (2016) 30:411–20. 10.1016/j.blre.2016.05.001 27238576

[6] Rouphael NG Talati NJ Vaughan C Cunningham K Moreira R Gould C. Infections associated with haemophagocytic syndrome. Lancet Infect Dis. (2007) 7:814–22. 10.1016/S1473-3099(07)70290-6 18045564 PMC7185531

[7] Zhou M Li L Zhang Q Ma S Sun J Zhu L. Clinical features and outcomes in secondary adult hemophagocytic lymphohistiocytosis. QJM. (2018) 111:23–31. 10.1093/qjmed/hcx183 29025045

[8] Ishii E Ohga S Imashuku S Yasukawa M Tsuda H Miura I. Nationwide survey of hemophagocytic lymphohistiocytosis in Japan. Int J Hematol. (2007) 86:58–65. 10.1532/IJH97.07012 17675268

[9] Soy M Atagunduz P Atagunduz I Sucak GT. Hemophagocytic lymphohistiocytosis: a review inspired by the COVID-19 pandemic. Rheumatol Int. (2021) 41:7–18. 10.1007/s00296-020-04636-y 32588191 PMC7315691

[10] Henter JI Samuelsson-Horne A Arico M Egeler RM Elinder G Filipovich AH. Treatment of hemophagocytic lymphohistiocytosis with HLH-94 immunochemotherapy and bone marrow transplantation. Blood. (2002) 100:2367–73. 10.1182/blood-2002-01-0172 12239144

[11] Otrock ZK Grossman BJ Eby CS. Transfusion requirements and 30-day mortality predictors for adult hemophagocytic lymphohistiocytosis. Int J Hematol. (2018) 108:485–90. 10.1007/s12185-018-2504-5 30043331

[12] Knaak C Nyvlt P Schuster FS Spies C Heeren P Schenk T. Hemophagocytic lymphohistiocytosis in critically ill patients: diagnostic reliability of HLH-2004 criteria and HScore. Crit Care. (2020) 24:244. 10.1186/s13054-020-02941-3 32448380 PMC7245825

[13] Ramos-Casals M Brito-Zeron P Lopez-Guillermo A Khamashta MA Bosch X. Adult haemophagocytic syndrome. Lancet. (2014) 383:1503–16. 10.1016/S0140-6736(13)61048-X 24290661

[14] Li J Wang Q Zheng W Ma J Zhang W Wang W. Hemophagocytic lymphohistiocytosis: clinical analysis of 103 adult patients. Medicine. (2014) 93:100–5. 10.1097/MD.0000000000000022 24646466 PMC4616310

[15] Huang J Yin G Duan L Tian T Xu J Wang J. Prognostic value of blood-based inflammatory biomarkers in secondary hemophagocytic lymphohistiocytosis. J Clin Immunol. (2020) 40:718–28. 10.1007/s10875-020-00801-x 32495220

[16] Zhou J Zhou J Wu ZQ Goyal H Xu HG. Ferritin index is a strong prognostic marker in adult hemophagocytic lymphohistiocytosis. Int J Clin Pract. (2020) 75:e13704. 10.1111/ijcp.13704 32931059

[17] Zhou J Zhou J Wu ZQ Goyal H Xu HG. A novel prognostic model for adult patients with Hemophagocytic Lymphohistiocytosis. Orphanet J Rare Dis. (2020) 15:215. 10.1186/s13023-020-01496-4 32819431 PMC7439554

[18] Pei R Wang Z Wang Y Shi X Zhang R Zheng H. [A multicenter retrospective etiological analysis of 601 patients with hemophagocytic lymphohistiocytosis in China]. Zhonghua Nei Ke Za Zhi. (2015) 54:1018–22. 10.3760/cma.j.issn.0578-1426.2015.12.006 26887367

[19] Griffin G Shenoi S Hughes GC. Hemophagocytic lymphohistiocytosis: an update on pathogenesis, diagnosis, and therapy. Best Pract Res Clin Rheumatol. (2020) 34:101515. 10.1016/j.berh.2020.101515 32387063

[20] Hou H Luo Y Wang F Yu J Li D Sun Z. Evaluation of lymphocyte function by IFN-gamma secretion capability assay in the diagnosis of lymphoma-associated hemophagocytic syndrome. Hum Immunol. (2019) 80:1006–11. 10.1016/j.humimm.2019.09.003 31540793

[21] Brito-Zeron P Kostov B Moral-Moral P Martinez-Zapico A Diaz-Pedroche C Fraile G. Prognostic factors of death in 151 adults with hemophagocytic syndrome: etiopathogenically driven analysis. Mayo Clin Proc Innov Qual Outcomes. (2018) 2:267–76. 10.1016/j.mayocpiqo.2018.06.006 30225460 PMC6132215

[22] Cui Y Xiong X Ren Y Wang F Wang C Zhang Y. CD163 as a valuable diagnostic and prognostic biomarker of sepsis-associated hemophagocytic lymphohistiocytosis in critically ill children. Pediatr Blood Cancer. (2019). 66:e27909. 10.1002/pbc.27909 31298489

[23] Liu LL Qiu HX Xu J Duan LM Tian T Wang JJ. [The clinical significance of plasma PTX3 in patients with secondary hemophagocytic lymphohistiocytosis]. Zhonghua Nei Ke Za Zhi. (2020) 59:528–34. 10.3760/cma.j.cn112138-20191112-00745 32594686

[24] Nanno S Koh H Katayama T Hashiba M Sato A Makuuchi Y. Plasma levels of presepsin (soluble CD14-subtype) as a novel prognostic marker for hemophagocytic syndrome in hematological malignancies. Intern Med. (2016) 55:2173–84. 10.2169/internalmedicine.55.6524 27522992

[25] Yoon JH Park SS Jeon YW Lee SE Cho BS Eom KS. Treatment outcomes and prognostic factors in adult patients with secondary hemophagocytic lymphohistiocytosis not associated with malignancy. Haematologica. (2019) 104:269–76. 10.3324/haematol.2018.198655 30213834 PMC6355492

[26] Li DL Shen F Yin Y Peng JX Chen PY. Weighted Youden index and its two-independent-sample comparison based on weighted sensitivity and specificity. Chin Med J. (2013) 126:1150–4. 10.3760/cma.j.issn.0366-6999.20123102 23506596

[27] Jordan MB Allen CE Greenberg J Henry M Hermiston ML Kumar A. Challenges in the diagnosis of hemophagocytic lymphohistiocytosis: recommendations from the North American Consortium for Histiocytosis (NACHO). Pediatr Blood Cancer. (2019) 66:e27929. 10.1002/pbc.27929 31339233 PMC7340087

