# Supplementary figures and images for: Clinical characteristics and risk factors for 90-day overall survival among 204 adult patients with secondary hemophagocytic lymphohistiocytosis: Experience from a single-center retrospective study
Source: Front Med (Lausanne). 2022 Oct 10;9:774959. doi: 10.3389/fmed.2022.774959 (PMC9589347; doi:10.3389/fmed.2022.774959)

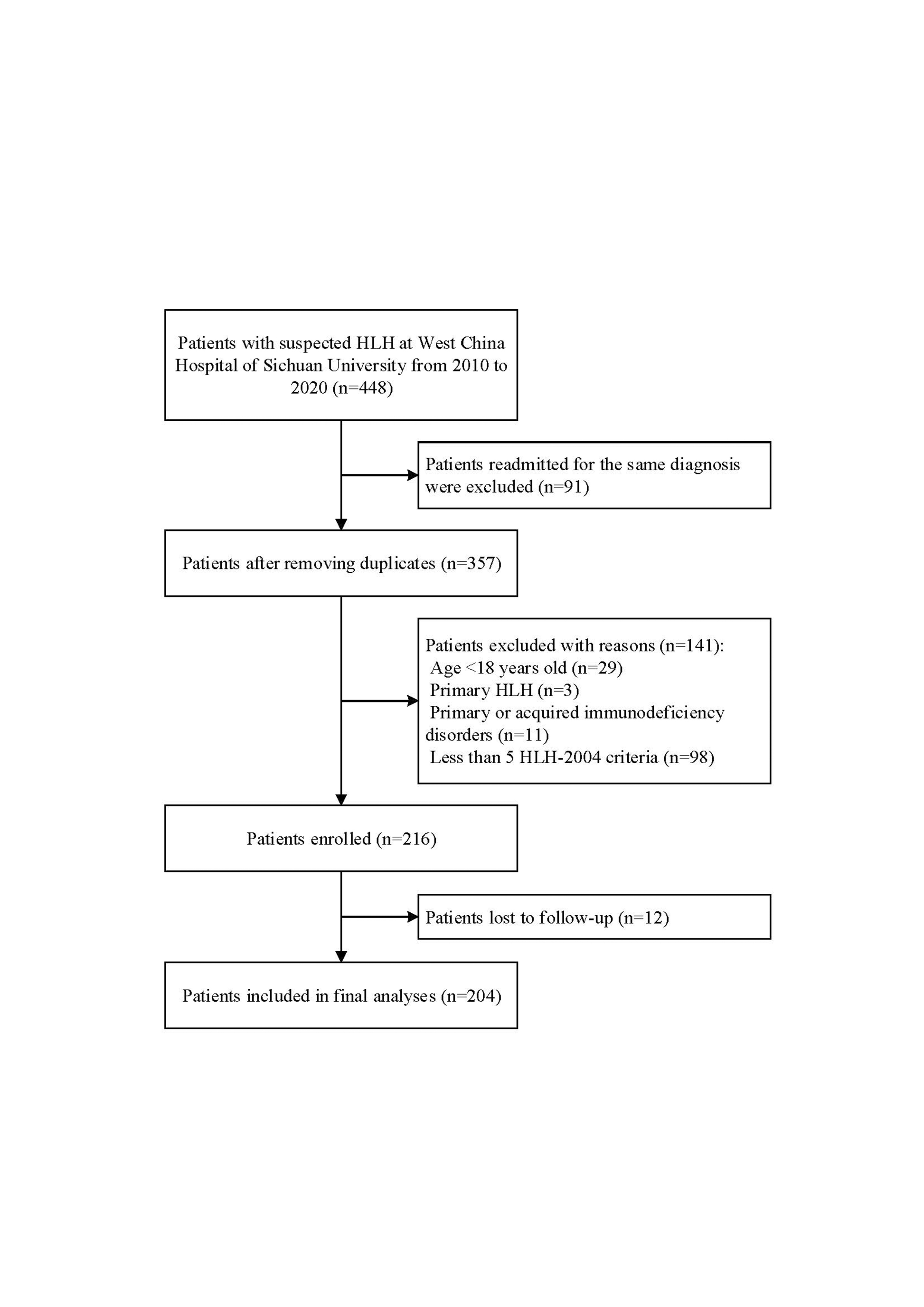

Supplement: Supplementary Figure 1 — Flow chart of included patients and the simplified model. [file Image_1.jpg]
