# Supplementary material for: Clinical characteristics and risk factors for 90-day overall survival among 204 adult patients with secondary hemophagocytic lymphohistiocytosis: Experience from a single-center retrospective study
Source: Front Med (Lausanne). 2022 Oct 10;9:774959. doi: 10.3389/fmed.2022.774959 (PMC9589347; doi:10.3389/fmed.2022.774959)
Supplement: Supplementary Table 1 — Comparison of clinical characteristics between survivors and non-survivors of secondary HLH with different causes. [file Table_1.pdf]

Supplementary table 1. Comparison of clinical characteristics between survivors and non-survivors of secondary HLH with different causes.

	Infections (n=65)			Malignancies (n=125)			Autoimmune disorders (n=8)			Unknown causes (n=6)		
	Survivors (n=44)	Non-survivors (n=21)	*P* value	Survivors (n=79)	Non-survivors (n=46)	*P* value	Survivors (n=6)	Non-survivors (n=2)	*P* value	Survivors (n=5)	Non-survivors (n=1)	*P* value
Splenomegaly	33(75.0)	11(52.4)	0.068	77(97.5)	39(84.8)	**0.008***	6(100.0)	2(100.0)	-	4(80.0)	1(100.0)	1.000
Coagulopathy	7(15.9)	10(47.6)	**0.007***	16(20.3)	17(37.0)	**0.041***	1(16.7)	0(0)	1.000	2(40.0)	0(0)	1.000
Hemoglobin (g/L)	79.00(68.25,99.75)	78.00(61.00,86.50)	0.055	83.00(71.00,94.00)	72.00(60.75,78.75)	**<0.001***	78.50(66.00,91.50)	74.00(67.00,-)	0.728	72.00(62.50,89.50)	82.00	-
Platelet ($\times10^9$/L)	48.00(29.25,88.00)	29.00(15.50,48.50)	**0.014***	43.00(18.00,69.00)	29.00(14.75,50.25)	**0.040***	81.50(36.00,139.25)	29.50(12.00,-)	0.429	34.00(21.50,97.00)	10.00	0.333
TB (μmol/L)	12.70(8.88,23.43)	30.60(11.85,91.35)	**0.010***	17.20(11.30,36.60)	24.20(15.90,75.78)	**0.025***	12.70(6.10,39.65)	84.45(53.10,-)	0.143	78.00(24.75,108.45)	223.30	0.333
ALB (g/L)	27.35(24.63,30.75)	26.40(22.60,30.40)	0.210	28.50(25.10,32.70)	26.85(23.10,30.33)	**0.012***	27.65(24.55,32.93)	24.95(22.30,-)	0.390	24.40(22.70,25.15)	22.00	-
LDH (IU/L)	642.50(439.50,1160.25)	1166.00(598.50,2731.00)	**0.037***	589.00(375.00,1135.00)	892.00(673.50,1458.50)	**0.003***	719.50(475.75,892.25)	1059.50(1008.00,-)	0.286	609.00(292.50,1985.00)	824.00	1.000
BUN (mmol/L)	4.80(3.33,7.78)	7.30(4.52,14.71)	**0.006***	5.17(4.04,7.50)	6.11(4.75,8.33)	0.118	3.95(2.28,6.85)	5.35(5.20,-)	0.643	3.90(3.15,6.21)	17.27	0.333
TG (mmol/L)	1.92(1.2	3.00(2.0	**0.011***	2.56(1.6	3.05(2.1	0.105	2.82(1.5	6.45(4.1	0.071	4.01(2.9	5.51	0.667

	8,2.94)	9,4.15)		5,3.80)	3,4.23)		7,3.44)	1,-)		7,7.57)		
FIB (g/L)	1.41(1.18,2.84)	0.75(0.61,1.13)	**<0.001***	1.30(0.81,2.32)	1.01(0.68,1.31)	**0.023***	1.46(1.06,1.81)	1.05(0.53,-)	0.857	1.67(0.72,2.36)	0.89	1.000

$^*P$<0.05.

Abbreviations: TB, total bilirubin; ALB, albumin; ALB, albumin; LDH, lactate dehydrogenase; BUN, blood urea nitrogen; TG, triglyceride; FIB, fibrinogen.

The laboratory results were those obtained on the day of diagnosis of hemophagocytic lymphohistiocytosis, or those obtained up to three days before or after in the absence of data from the same day.

Supplementary table 2. Clinical and laboratory findings of included secondary HLH patients according to the HLH-2004 criteria.

Parameter	No. of patients (%)
Fever (≥38.5℃)	191/204(93.6)
Splenomegaly	173/204(84.8)
Two lineage of cytopenia or pancytopenia (hemoglobin <90g/L, platelets $<100\times10^9$/L, neutrophils $<1.0\times10^9$/L)	174/204(85.3)
Hypertriglyceridemia (triglyceride >3.0mmol/L) and/or hypofibrinogenia (fibrigen <1.5g/L)	164/204(80.4)
Hemophagocytosis found in bone marrow/spleen/lymph nodes	87/204(42.6)
Hyperferritinemia (ferritin ≥500μg/L)	194/196(99.0)
Elevated concentration of soluble interleukin 2 receptor (sIL-2R/sCD25 ≥2400u/mL)	128/137(93.4)
Low or absent activity of natural killer cells	NA

Abbreviations: HLH, hemophagocytic lymphohistiocytosis; NA, not available.
